# Effect of Black Maca Supplementation on Inflammatory Markers and Physical Fitness in Male Elite Athletes

**DOI:** 10.3390/nu15071618

**Published:** 2023-03-27

**Authors:** Eunjae Lee, Myeonghun Park, Byoungju Kim, Sunghwun Kang

**Affiliations:** 1 Institute of Sports & Arts Convergence (ISAC), Inha University, Incheon 22212, Republic of Korea; eunjaesports@gmail.com; 2 Waseda Institute for Sport Sciences, Waseda University, Saitama 341-0018, Japan; 3 Charmacist, Seoul 02797, Republic of Korea; 24kpurity@charmacist.com (M.P.); ceo@charmacist.com (B.K.); 4 Laboratory of Exercise Physiology, College of Art, Culture and Engineering, Kangwon National University, Chuncheon-si 24341, Republic of Korea; 5 Interdisciplinary Program in Biohealth-Machinery Convergence Engineering, Kangwon National University, Chuncheon-si 24341, Republic of Korea

**Keywords:** black maca, supplementation, inflammation, physical fitness, athletes

## Abstract

Given the current lack of studies, the primary purpose of this study was to investigate the effects of black maca supplementation intake on changes in physical strength and inflammatory markers among elite athletes. Forty-four elite athletes were recruited for the present study. They included shooting athletes, racket sports athletes, and fin swimming athletes. The intake capsules contained 2500 mg of 100% concentrated black maca extract. Participants were instructed to take one capsule twice a day for eight weeks with pure water. Changes were seen in the ATP-PC systems and aerobic energy systems, particularly in the fin swimming athletes requiring aerobic energy systems. This effect is caused by increased antioxidant activity and influenced mitochondrial biosynthesis regulatory factors due to black maca supplementation intake. These findings provide preliminary evidence that elite athletes will benefit from taking black maca to improve their inflammation levels and physical fitness.

## 1. Introduction

The typical reasons for an athlete’s use of supplements include improving overall health conditions, improving cognitive or physical performance, increasing energy levels, reducing excessive weight, relieving pain, and other favorable effects [1]. In addition, athletes frequently include supplements in their diet to compensate for any nutritional deficiencies [2]. For various reasons, elite athletes commonly take various supplements to improve their performance.

Maca (Lepidium meyenii) is a plant that grows in the central Andes region of Peru, at an elevation of more than 4000 m, and contains a variety of species depending on the color of the hypocotyl and differences between the effects of black, yellow, and red maca varieties [3]. Maca is rich in fiber, and contains many essential amino acids, fatty acids, and other nutrients, including vitamin C, copper, iron, and calcium [4]. The darker the color, the better the quality, and the higher the active ingredient [5]. Among them, black maca exhibits an increased antioxidant ability [6], sperm count [7], cell protection [8], and endurance [3] than the other varieties. Maca is prepared and provided in the form of a capsule or powder, as a natural component. The chemical composition of maca is primarily responsible for its biological and pharmacological activities [9]. 

A previous study reported that taking maca-treatment and participating in a daily 30-min swim over a 28-day period increased the maximum swimming time and had long-term stamina-enhancing effects and significantly recovered blood parameters of energy, as well as muscular in male mice [10]. In addition, several previous studies have reported the benefits for athletes of taking maca. A 14-day maca supplement greatly improved the time to complete a 40 km time test and increased the self-reported sexual desire in trained male cyclists [11]. After female elite athletes completed a four-week course of black maca supplement, results showed decreased C-reactive protein concentrations and increased endurance of trunk muscles [12]. A 16-week course of black maca supplement in university racket athletes participating in resistance exercises showed lowered blood ammonia levels and improved muscle function [13].

A study by Yu et al. suggested that the effect of maca extracts on mitochondria in spinal cells and that the maca extracts may improve muscle antioxidant activity by raising levels [14]. Although this support may help elite athletes improve their performance, studies that observe the physiological changes in elite athletes taking black maca supplementation are still insufficient. The primary purpose of this study was to investigate the intake of black maca supplementation and examine changes in physical strength and the plasma components of elite athletes.

## 2. Methods

### 2.1. Participation

Forty-four male elite athletes were recruited for the present study. They included shooting athletes (SA, *n* = 15), racket sports athletes (soft tennis and table tennis) (RSA, *n* = 16), and fin swimming athletes (FSA, *n* = 13). There were no significant differences in age, height, weight, BMI, and fat percentage.

The participants were fully informed about the study’s objectives, methodology, and goals to ensure their complete understanding. This study adhered to the ethical standards outlined in the Declaration of Helsinki, and all participants signed an informed consent form prior to their participation. Additionally, the Kangwon National University Review Board for Human Subjects approved this study (KWNUIRB-2021-04-013-002).

Table 1 lists the physical characteristics of the participants.

### 2.2. Measurement of Body Composition

The body composition variables of the participants were measured. Using a body composition analyzer (Inbody 720, Body Composition Analyzer; Biospace, Seoul, Republic of Korea), weight, height, and body fat percentage (as a percentage) were measured. The body mass index (BMI) was determined by dividing weight in kilograms by the square of height in meters.

### 2.3. Hematological Analysis

At the baseline and eight-week, all participants were required to fast for 12 h before venous blood samples were taken. The following day, blood samples were collected at 8:30 a.m. from the veins after ensuring adequate sleep and minimizing movement. After collection, the samples were immediately centrifuged at 3500× *g* for 10 min at 4 °C, and the serum was stored at −80 °C for further analysis. Enzyme-linked immunosorbent assay (ELISA) kits from DueSet™ (R&D systems, Minneapolis, MN, USA) were used to measure plasma levels of myoglobin, creatine kinase (CK), lactic acid, total cholesterol (TC), high density lipoprotein cholesterol (HDL-C), C-reactive protein (CRP), interleukin-6 (IL-6), and tumor necrosis factor-alpha (TNF-α), following the manufacturer’s instructions as previously described.

### 2.4. Measurement of Physical Fitness

All participants were evaluated for physical fitness variables at the baseline and eight weeks. The evaluation included measurements of muscle strength, muscle endurance, flexibility, power, agility, cardiovascular endurance, and isokinetic trunk muscle function.

To evaluate muscle strength (kg), a digital grip dynamometer (GRIP-D 5101; TAKEI, Co., Tokyo, Japan) was used to measure grip strength. Muscle endurance (number of repetitions/1 min) was evaluated using sit-ups (BS-SU Inbody, Seoul, Republic of Korea), while flexibility (cm) was assessed using sit and reach exercises (TKK-5403; TAKEI, Co., Tokyo, Japan). Power (cm) was evaluated using a long jump, and agility (s) was assessed using a 10 m shuttle run. Cardiovascular endurance (repetition) was assessed using a 20 m shuttle run, as previously described by Kang et al. [15]. Each assessment for muscle strength, muscle endurance, flexibility, power, and agility was performed twice, and the highest score was retained.

Isokinetic dynamometer machines (Humac Norm Testing and Rehabilitation, CSMi Medical & Solution, Stoughton, MA, USA) were used to measure the strength and endurance of the trunk muscles. Before data collection, isokinetic dynamometers were calibrated according to the manufacturer’s instructions. After calibration, subjects were positioned on the isokinetic dynamometers for assessment according to the manufacturer’s recommendations. The participants performed warm-up exercises, in the form of dynamic stretching for trunk flexion and extension, before measurements were recorded. The maximum isokinetic trunk strength was measured three times at 30°/s, and trunk endurance was measured three times at 120°/s. The range of trunk motion during the tests was set from −10° to 70°.

### 2.5. Taking Black Maca Supplementation

The intake capsules contained 2500 mg of 100% concentrated black maca extract. The black maca constituents included fiber (4.95 g/100 g), carbohydrate (63.82 g/100 g), protein (7.7 g/100 g), starch (38.18 g/100 g), soluble sugar (7.02 g/100 g), riboflavin (0.76 mg/100 g), potassium (1000 mg/100 g), and iron (86 mg/100 g). The capsules included crystalline cellulose, starch, calcium stearate, and silicon dioxide as a filler. The capsules were of a deep cooler and the participants could not see the contents. Participants were instructed to take one capsule twice a day for eight weeks with pure water. The dosage time was in the morning and evening; however, the specific time was not designated.

### 2.6. Statistical Analysis

All results are reported as the mean ± standard deviation. All data were analyzed using SPSS version 25.0 (SPSS Inc., Chicago, IL, USA). First, the one-way ANOVA was used to assess group differences in baseline variables. For the three groups (shooting athletes (SA), racket sports athletes (RSA), and fin swimming athletes (FSA)) by two stages (baseline and eight weeks), a two-way factor ANOVA was used to examine whether taking black maca supplementation had any effect. A Bonferroni test was used for the post hoc analysis. Second, paired *t*-tests were used to compare the baseline and post-taking black maca supplementation (eight weeks) performances of the same groups. Statistical significance was accepted at a = 0.05.

## 3. Results

### 3.1. Change in Physical Fitness after Taking Black Maca Supplementation

Changes in the physical fitness of the participants in each group are shown in Table 2. Two-way within-factor ANOVA revealed significant group × time interaction for left and right grip muscle strength and long jump power (*p* < 0.05, *p* < 0.01, respectively). The paired *t*-test analysis showed a significant increase in muscle endurance (*p* < 0.01) and agility (*p* < 0.05) in the SA group; muscle endurance (*p* < 0.05), power (*p* < 0.05) and agility (*p* < 0.05) in the RSA group; and muscle strength (*p* < 0.01), muscle endurance (*p* < 0.01), flexibility (*p* < 0.01), power (*p* < 0.001) and agility (*p* < 0.001) in the FSA group after taking black maca supplementation for eight weeks. 

### 3.2. Change in Isokinetic Muscle Function of Trunk after Taking Black Maca Supplementation

Changes in muscle function of the trunk in each group are shown in Table 3. There was no significant group × time interaction. Post hoc analysis using the Bonferroni test indicated that 30°/s extensor in the RSA group was significantly higher, and 120°/s extensor in the SA group was significantly lower after taking black maca supplementation for 8 weeks. The paired *t*-tests analysis showed a significant increase in 120°/s extensor (*p* < 0.05) in the FSA group and 120°/s flexor (*p* < 0.01) in the SA group after taking black maca supplementation for 8 weeks. 

### 3.3. Change in Plasma Components after Taking Black Maca Supplementation

Changes in the plasma components in each group are shown in Table 4. Two-way within-factor ANOVA revealed a significant group × time interaction for lactic acid and TC (*p* < 0.01, *p* < 0.001, respectively). Post hoc analysis using the Bonferroni test indicated that lactic acid, TC, and CRP in the SA group were significantly higher after taking black maca supplementation for eight weeks. The paired *t*-test analysis showed a significant difference in lactic acid (*p* < 0.05) and TC (*p* < 0.01) in the SA group; TC (*p* < 0.01) and CRP (*p* < 0.01) in the RSA group; and lactic acid (*p* < 0.05), TC (*p* < 0.001), and CRP (*p* < 0.001) in the FSA group after taking black maca supplementation for eight weeks. 

### 3.4. Change in Inflammation after Taking Black Maca Supplementation

Changes in inflammation in each group are shown in Table 5. There was no significant group × time interaction. Post hoc analysis using the Bonferroni test indicated that IL-6 in the FSA group was significantly higher than in the other groups after taking black maca supplementation for eight weeks. The paired *t*-test analysis showed a significant decrease in IL-6 (*p* < 0.001) and TNF-α (*p* < 0.01) in the RSA group and TNF-α (*p* < 0.01) in the FSA group after taking black maca supplementation for 8 weeks. 

## 4. Discussion

In this study, changes in inflammatory markers and physical fitness, after taking black maca supplementation for eight weeks, were investigated. The main finding of this study was that IL-6, TNF-α, TC, and CRP levels were significantly different after taking black maca supplementation in the RSA group; the TNF-α, lactic acid, TC, and CRP levels were significantly different after taking black maca supplementation in the FSA group; and lactic acid and TC were significantly different after taking black maca supplementation in the SA group. In addition, the muscle strength, muscle endurance, flexibility, power, and agility significantly increased after taking black maca supplementation in the FSA group. Muscle endurance significantly increased in the SA group. Muscle endurance, power, and agility significantly increased after taking black maca supplementation in the RSA group. 

Regular physical activity or exercise may induce long-term adaptations in metabolism and cardiovascular systems, including reactive oxygen species and proinflammatory cytokines reduction, which involve an increase in antioxidants and anti-inflammatory factors [16]. However, elite athletes who exercise repeatedly at high intensity are known to have an increased secretion of inflammatory cytokines, due to increased oxidation stress. A test of rowing up to 2000 m, in the shortest time possible on a rowing ergometer, was performed by twenty rowing athletes, and the results showed a significant increase in IL-6 and an increase in TNF, but the results were not statistically significant [17]. Fourteen athletes with spinal cord injuries (meter cervical spinal cord injuries and eight spinal cord injuries) participated in a wheelchair marathon. The results showed that the plasma IL-6 concentration increased significantly after the race, whereas plasma TNF-α concentrations showed no significant increase immediately after the race but a significant decrease after two hours [18]. Additionally, the significant correlations between overtraining syndrome related and levels of IL-6 and TNF-α have been reported in previous studies [19]. Exhaustion exercise reduces immune functions and releases inflammatory cytokines, which negatively affect an athlete’s performance [20]. In this study, it was shown that there were significant decreases in IL-6 (*p* < 0.001) and TNF-α (*p* < 0.01) in the RSA group and TNF-α (*p* < 0.01) in the FSA group after taking black maca supplementation for eight weeks. Extracts of maca contain saponins, phenols, and flavonoids and also have antioxidant activity (1,1-diphenyl-2-picrylhydrazyl radical scavenging activity and ferric-reducing ability of plasma) [21]. This antioxidant activity is also associated with lactic acid [22] and CRP [23]. Various endogenous antioxidant defense mechanisms exist, and antioxidant nutrients are consumed in the diet, which provide protection against the excessive production of reactive oxygen species (ROS) [24]. In this study, significant decreases in lactic acid (*p* < 0.05), TC (*p* < 0.001), and CRP (*p* < 0.001) were found in the FSA group after taking maca supplementation. In the SA group, only lactic acid (*p* < 0.05) showed a significant decrease, and in the RSA group, CRP (*p* < 0.01) significantly decreased, but TC (*p* < 0.01) significantly increased. There was a significant increase in TC in the SA and RSA groups but within the normal range. In all three groups, HDL-C tended to increase, but there was no statistically significant difference.

Continuous training will usually develop physical fitness, which, in turn, leads to better athletic performance [25]. Physical fitness differed among players of different sports [26]. Elite athletes have different physical fitness depending on the sports event, but they are improving through continuous training and taking supplements. Creatine monohydrates, vitamin D, omega-3 fatty acids, probiotics, gelatin/collagen, and certain anti-inflammatory supplements can affect the health and recovery of cells and tissues in a way that enables athletes to stay healthy, adapt to exercise, and increase the quality and amount of training [27]. Protein is one of the most popular dietary supplements for athletes and physically active people, and most amino acid supplements are safe at the recommended doses; however, excessive consumption may interfere with protein metabolism, and the World Anti-Doping Agency (WADA) does not ban the use of amino acid supplements [28]. Ikeda et al. reported that branched-chain amino acid intake with muscle-strengthening exercises had a significant effect on knee extension strength of the contralateral side and on upper arm cross-sectional area [29]. Hoffman et al. reported that participants in the above protein intake recommended group showed 22% and 42% greater changes in delta 1-RM squats and delta 1-RM bench presses than participants in the below protein intake recommended group in strength/power athletes [30]. In this study, natural black maca was taken for eight weeks and resulted in a significant increase in 120°/s extensor (*p* < 0.05) in the FSA group and 120°/s flexor (*p* < 0.01) in the SA group. Additionally, muscle strength (*p* < 0.01), muscle endurance (*p* < 0.01), flexibility (*p* < 0.01), power (*p* < 0.001), and agility (*p* < 0.001) were significantly increased in the FSA group after taking black maca supplementation for eight weeks. Earlier, the addition of arginine was mentioned as a component of black maca [31]. Arginine is an amino acid that has shown a vasodilator effect [32]. Arginine induces the expression of key regulatory factors of mitochondrial biosynthesis (PGC-1α; proliferator-activated receptor gamma coactivator-1α) and the genes encoding complex I. It can also increase the production of nitrogen monoxide and maximize oxygen consumption [33]. Although there was no difference in most variables in the isokinetic trunk muscle function test, there was a significant increase in all items, except for cardiopulmonary endurance, for physical fitness in the FSA group. In this study, although there was no treatment other than taking black maca supplementation, the improvement of the athlete’s physical fitness is believed to be due to the presence of arginine in the black maca. In cardiopulmonary endurance, due to the characteristics of the FSA group, it is believed that it has been adapted to continuous aerobic training. In addition, muscle endurance and agility were significantly increased in the SA group, and muscle endurance, power, and agility were significantly increased in the RSA group. Traditionally, ATP-PC systems operate without oxygen and glycolysis uses 2–3 min of energy as a source of glucose energy, so glycolysis does not need oxygen, and the aerobic energy system continues to use oxygen to supply energy during training or competition occurring over a long period of time [34]. Therefore, considering the sports event and characteristics of the participants in this study, the SA and RSA groups consisted of lower-intensity training athletes than the FSA groups, which would have required more energy from ATP-PC systems and glycolysis processes than aerobic training. 

The present study had some limitations. The sample size was small. Future studies with larger sample sizes and a control group are required to consolidate the effectiveness of black maca supplementation on changes in plasma components and physical fitness in athletes. Another limitation was that we did not control for contributing factors such as habitual activity and food intake. We assumed that participants lived in the same dormitory and had the same diet throughout the study period. However, habitual activity and food energy intake should be considered in future studies. Finally, the study period was short; therefore, more longitudinal studies are required to investigate the effects of taking black maca supplementation.

## 5. Conclusions

In conclusion, this study indicated that taking black maca supplementation changed the levels of inflammation and plasma components such as IL-6, TNF-α, lactic acid, TC, and CRP. Additionally, taking black maca supplementation was effective in improving physical fitness. The decrease in inflammation and improvement of physical fitness in elite athletes helped their performance. These changes were observed in the requirement of ATP-PC and aerobic energy systems but more in fin swimming athletes (FSA) requiring aerobic energy systems. This effect is the increased antioxidant activity and influenced mitochondrial biosynthesis and associated regulatory factors due to black maca supplementation intake. When the aerobic metabolism of mitochondria is dominant, mitochondrial biosynthesis and antioxidant defense systems are regulated to prevent the accumulation of ROS [35]. These findings provide preliminary evidence that elite athletes need to take black maca supplementation to improve their inflammation levels and physical fitness.

## Figures and Tables

**Table 1:** The characteristic of the participants.

Variable	SA (*n* = 15)	RSA (*n* = 16)	FSA (*n* = 13)
Age (years)	27.07 ± 6.33	21.25 ± 1.30	22.54 ± 3.13
Height (cm)	174.5 ± 5.45	176.7 ± 4.14	169.6 ± 4.96
Weight (kg)	76.89 ± 9.74	71.28 ± 9.72	70.79 ± 8.82
BMI (kg/m^2^)	25.20 ± 2.43	22.80 ± 3.06	24.50 ± 2.01
% fat (%)	22.71 ± 6.66	17.34 ± 4.91	22.04 ± 8.35

Values are mean (SD). BMI, body mass index; SA, shooting athletes; RSA, racket sports athletes; FSA, fin swimming athletes.

**Table 2:** Measurements of physical fitness parameters by group and time.

Variable	Time	Group			*p*-Value	Post Hoc
		SA (*n* = 13) ^a^	RSA (*n* = 15) ^b^	FSA (*n* = 13) ^c^		
Left grip strength (kg)	Pre	43.8 ± 4.7	41.0 ± 5.9	34.9 ± 7.5	G: 0.012 T: 0.008 GxT: 0.037	b > c
	Post	45.1 ± 4.0	41.0 ± 5.0	39.0 ± 9.9 **		
Right grip strength (kg)	Pre	46.0 ± 5.2	46.2 ± 5.8	38.1 ± 8.1	G: 0.026 T: 0.002 GxT: 0.032	b > c
	Post	47.0 ± 6.3	46.8 ± 5.4	42.1 ± 9.8 **		
Sit-ups (rep)	Pre	40.2 ± 7.0	41.5 ± 9.3	55.8 ± 11.2	G: <0.001 T: <0.001 GxT: 0.179	c > a, b
	Post	47.1 ± 7.7 **	47.7 ± 8.0 *	57.9 ± 8.7 **		
Sit-and-reach (cm)	Pre	8.53 ± 8.4	6.91 ± 11.4	22.2 ± 9.5	G: <0.001 T: <0.001 GxT: 0.720	c > a, b
	Post	10.4 ± 7.2	9.9 ± 11.1	25.3 ± 8.1 **		
Long jump (cm)	Pre	210.5 ± 19.2	228.9 ± 14.0	197.8 ± 33.5	G: 0.007 T: <0.001 GxT: 0.006	b > a, c
	Post	215.0 ± 15.1	239.5 ± 12.8 *	222.1 ± 32.5 ***		
10 m shuttle run (s)	Pre	10.0 ± 0.6	9.1 ± 0.7	10.0 ± 0.9	G: <0.001 T: <0.001 GxT: 0.106	b < a, c
	Post	10.5 ± 0.6 *	9.5 ± 0.4 *	11.0 ± 1.2 ***		
20 m shuttle run (rep)	Pre	33.3 ± 3.6	62.9 ± 9.4	74.5 ± 19.3	G: <0.001 T: 0.814 GxT: 0.171	a < b, c
	Post	37.5 ± 8.0	64.1 ± 12.6	70.4 ± 17.1		

Values are expressed as Mean ± SD. SA, shooting athletes; RSA, racket sports athletes; FSA, fin swimming athletes. a = SA group, b = RSA group, c = FSA group. Analyzed by paired *t*-test: * *p* < 0.05, ** *p* < 0.01, and *** *p* < 0.001.

**Table 3:** Measurements of isokinetic muscle function of trunk parameters by group and time.

Variable	Time	Group			*p*-Value	Post Hoc
		SA (*n* = 13) ^a^	RSA (*n* = 15) ^b^	FSA (*n* = 13) ^c^		
30°/s Extensor (%BW)	Pre	272.1 ± 56.2	340.1 ± 34.8	284.8 ± 86.6	G: 0.002 T: 0.131 GxT: 0.329	b > a, c
	Post	274.8 ± 47.3	322.0 ± 63.3	241.6 ± 64.0		
30°/s Flexor (%BW)	Pre	282.9 ± 31.8	307.8 ± 39.8	315.2 ± 36.7	G: 0.086 T: 0.269 GxT: 0.054	-
	Post	290.7 ± 31.5	314.8 ± 56.3	261.7 ± 71.3		
120°/s Extensor (%BW)	Pre	158.6 ± 70.8	239.4 ± 67.6	234.9 ± 89.8	G: 0.002 T: 0.002 GxT: 0.780	a < b, c
	Post	190.1 ± 63.1	283.3 ± 71.1	287.6 ± 98.0 *		
120°/s Flexor (%BW)	Pre	275.7 ± 88.4	320.4 ± 102.0	317.6 ± 56.7	G: 0.249 T: 0.010 GxT: 0.586	-
	Post	327.9 ± 69.0 **	366.3 ± 62.8	335.8 ± 75.3		

Values are expressed as Mean ± SD. SA, shooting athletes; RSA, racket sports athletes; FSA, fin swimming athletes. a = SA group, b = RSA group, c = FSA group. Analyzed by paired *t*-test: * *p* < 0.05, and ** *p* < 0.01.

**Table 4:** Measurements of plasma components parameters by group and time.

Variable	Time	Group			*p*-Value	Post Hoc
		SA (*n* = 13) ^a^	RSA (*n* = 15) ^b^	FSA (*n* = 13) ^c^		
Myoglobin (mg/mL)	Pre	31.4 ± 17.5	30.0 ± 12.0	26.2 ± 6.6	G: 0.246 T: 0.413 GxT: 0.928	-
	Post	28.6 ± 8.6	29.3 ± 11.7	24.1 ± 5.0		
Creatine kinase (U/L)	Pre	439.3 ± 523.5	265.9 ± 234.4	313.2 ± 170.5	G: 0.215 T: 0.341 GxT: 0.808	-
	Post	327.4 ± 259.3	229.1 ± 202.3	289.8 ± 160.7		
Lactic acid (mg/dL)	Pre	15.1 ± 6.6	8.0 ± 2.5	11.1 ± 5.3	G: 0.001 T: 0.004 GxT: 0.004	a > b, c
	Post	10.4 ± 3.2 *	9.6 ± 3.6	6.5 ± 1.7 *		
TC (mg/dL)	Pre	174.6 ± 30.7	148.5 ± 24.2	170.5 ± 14.9	G: 0.001 T: 0.017 GxT: <0.001	a > b, c
	Post	200.3 ± 33.2 **	167.0 ± 21.1 **	151.5 ± 19.8 ***		
HDL-C (mg/dL)	Pre	54.9 ± 10.3	58.2 ± 11.0	59.7 ± 12.5	G: 0.741 T: 0.269 GxT: 0.178	-
	Post	60.4 ± 13.0	58.5 ± 13.1	61.2 ± 15.6		
CRP (mg/dL)	Pre	0.12 ± 0.11	0.08 ± 0.10	0.09 ± 0.02	G: 0.027 T: 0.001 GxT: 0.699	a > b
	Post	0.09 ± 0.09	0.02 ± 0.03 **	0.04 ± 0.01 ***		

Values are expressed as Mean ± SD. TC, total cholesterol; HDL-C, high density lipoprotein cholesterol; RP, C-reactive protein; SA, shooting athletes; RSA, racket sports athletes; FSA, fin swimming athletes. a = SA group, b = RSA group, c = FSA group. Analyzed by paired *t*-test: * *p* < 0.05, ** *p* < 0.01, and *** *p* < 0.001.

**Table 5:** Measurements of inflammation by group and time.

Variable	Time	Group			*p*-Value	Post Hoc
		SA (*n* = 13) ^a^	RSA (*n* = 15) ^b^	FSA (*n* = 13) ^c^		
IL-6 (pg/mL)	Pre	134.3 ± 6.38	136.9 ± 6.90	148.3 ± 18.9	G: 0.005 T: 0.003 GxT: 0.063	c > a, b
	Post	133.8 ± 2.96	132.0 ± 3.64 ***	146.3 ± 20.4		
TNF-α (pg/mL)	Pre	270.8 ± 26.6	278.1 ± 16.5	283.2 ± 22.2	G: 0.334 T: <0.001 GxT: 0.276	-
	Post	265.6 ± 11.7	265.6 ± 5.08 **	271.8 ± 16.6 **		

Values are expressed as Mean ± SD. IL-6, interleukin-6; TNF-α, tumor necrosis factor-alpha; SA, shooting athletes; RSA, racket sports athletes; FSA, fin swimming athletes. a = SA group, b = RSA group, c = FSA group. Analyzed by paired *t*-test: ** *p* < 0.01, and *** *p* < 0.001.

## Data Availability

Data is contained within the article. The data presented in this study are available on request from the corresponding author.
